# Photobiomodulation therapy for mitigating severity of radiodermatitis in cancer patients undergoing radiotherapy: a scoping review

**DOI:** 10.1007/s00520-024-08944-y

**Published:** 2024-10-28

**Authors:** Devika Rao, Cliva Neha Dsouza, Smitha S Prabhu, Praveen Kumar, Vijendra Prabhu

**Affiliations:** 1 Photoceutics and Regeneration Laboratory, Centre for Microfluidics, Biomarkers, Photoceutics and Sensors (μBioPS), Department of Biotechnology, Manipal Institute of Technology, Manipal Academy of Higher Education, Manipal, 576104 Karnataka India; 2 Department of Biotechnology, Manipal Institute of Technology, Manipal Academy of Higher Education, Manipal, 576104 Karnataka India; 3 Department of Dermatology, Venereology and Leprosy, Kasturba Medical College Manipal, Manipal Academy of Higher Education, Manipal, 576104 Karnataka India

**Keywords:** Radiotherapy, Radiodermatitis, Photobiomodulation therapy, Cancer, Therapeutics

## Abstract

**Purpose:**

Radiodermatitis (RD) is an adverse effect of radiation therapy. RD can negatively impact quality of life and can also hinder treatment in cancer patients. Photobiomodulation therapy (PBMT) has the potential to treat RD at the cellular level, and it is more promising compared to other therapy alternatives. This review aims to examine the effectiveness of PBMT for the treatment and management of RD in cancer patients undergoing radiation therapy.

**Methods:**

The methodology followed for the review was based on the framework proposed by Arksey and O’Malley, and the extensions by Levac et al. This involved a literature search in Scopus, PubMed, Embase, and Cochrane without any time limit, for original articles on the basis of the inclusion criteria, i.e., studies focusing on the effectiveness of PBMT on RD in cancer patients undergoing radiation therapy as an anticancer treatment. The review has been reported on the basis of the PRISMA-ScR checklist.

**Results:**

A total of 14 studies were reviewed, of which only 2 (14.28%) studies reported no significant effect of PBMT on RD; the remaining studies reported positive outcomes (85.71%) with no adverse effects. Among studies with positive outcomes, PBMT has been shown to be beneficial in reducing the severity of RD. Furthermore, PBMT application has been studied as a preventive measure (35.71%), treatment and management (50%), and for both the prevention and cure of RD (14.29%).

**Conclusion:**

Overall, PBMT can be considered a reliable and effective treatment modality for reducing the severity of RD. However detailed studies related to the long-term effects of PBMT, its effect on pain intensity and quality of life (QoL) will aid in better assessment of the technique. More clinical trials with a broader sample size could also aid in fine-tuning the efficacy of PBMT treatment modalities.

## Introduction

Radiodermatitis (RD) is a common adverse effect of rigorous anticancer treatments, with approximately 95% of patients receiving radiotherapy (RT) experiencing various degrees of RD [1]. High ionizing radiation (2–50 Gy) delivered to cancer patients can cause oxidative stress and an inflammatory response, leading to acute radiation dermatitis (ARD), which commonly occurs in sites such as the neck, chest, and abdomen [2]. ARD symptoms can vary from dryness or red rashes to dry desquamation to more severe forms such as moist desquamation. RD progressively worsens within 90 days of radiation treatment, usually 2–3 days after the start of radiation [3]. This severity can necessitate a decrease in radiation doses or even a cessation of treatment, hindering the radiation treatment routine, and becoming an obstacle in treating cancer patients. Furthermore, the risk of developing RD during RT can be influenced by various factors, such as breast volume, body mass index (BMI), smoking, and health conditions such as diabetes [4]. An additional factor contributing to the increased likelihood of severe consequences of RD is individual radiosensitivity (IRS) and the psychological stress associated with radiation therapy [5, 6].

**Fig. 1:**
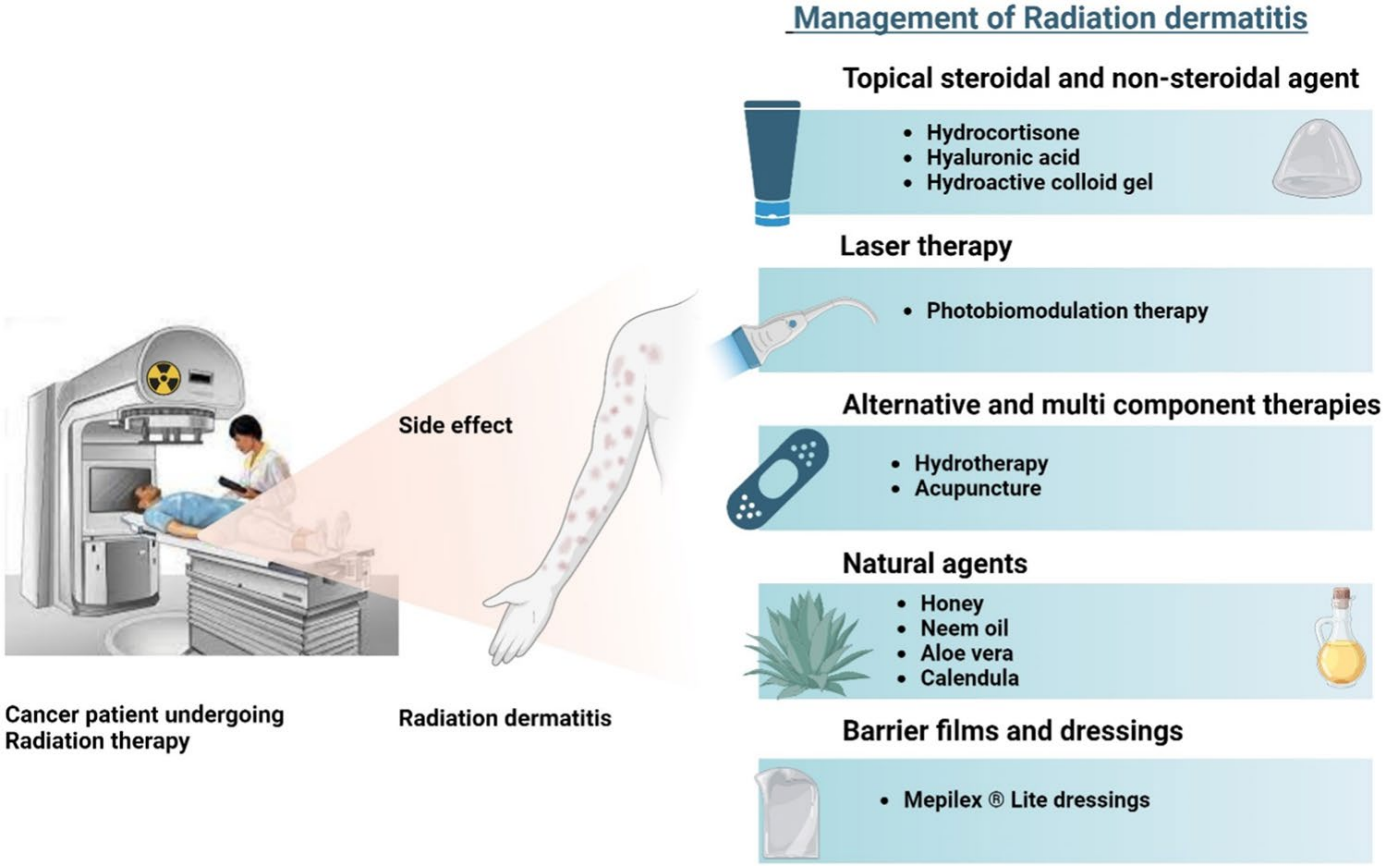
Current treatment options for the management of radiation dermatitis (created with BioRender.com)

Standard care for RD often involves maintaining hygiene by washing with a mild cleanser along with steroid application [7]. Although, no gold standard routine for the management of ARD has been established, various topical and systemic medications have been used to regulate radiation associated skin reactions. Figure 1 summarizes the current RD management options, which include topical corticosteroids [8], non-steroidal agents [9, 10], barrier films [11], natural agents [12–15], and multicomponent therapies [16, 17]. However, topical agents have limited efficiency and increased chances of adverse reactions [18]. Recent Multinational Association of Supportive Care in Cancer (MASCC) guidelines have recommended photobiomodulation therapy (PBMT) as an efficient method for preventing RD along with Mepitel ® film, Hydrofilm, olive oil, betamethasone, and mometasone [19].

Photobiomodulation therapy (PBMT), also known as low-level laser therapy, uses light in the wavelength range of 600–1100 nm, either from light emitting diodes (LEDs) or light amplification by stimulated emission of radiation (LASER), to elicit favorable cellular responses in biological tissue. The basic process includes stimulation of endogenous chromophores by absorption of light, leading to photochemical changes, resulting in proliferation, differentiation, and metabolic alterations [20]. PBMT treatments have been extensively explored for the treatment of acute wounds [21, 22], diabetic wounds [23], and thermal wounds [24] in various preclinical models. The application of PBMT as a supportive treatment for cancer patients has grown exponentially. PBMT has been effective in managing adverse effects of cancer therapy, including oral mucositis [25], lymphedema [26], and radiodermatitis [27]. In particular, the PBMT technique is used as complementary therapy in patients who have developed RD as a result of aggressive anticancer therapy such as RT. The purpose of the present study was to investigate whether PBMT is effective for the treatment and management of radiation dermatitis in cancer patients undergoing radiation therapy.

## Methodology

### Review question

We employed the PCC strategy to search for articles (Table 1). This study aimed to answer the following question: What is the effectiveness of PBMT treatment employed for the treatment and management of RD in cancer patients undergoing radiation therapy?

**Table 1:** PCC strategy employed for the article search

Population (P)	Cancer patients with radiodermatitis
Concept (C)	Application of photobiomodulation therapy (PBMT) in treating radiodermatitis
Context (C)	N/A

### Review design

The scoping review was conducted in five stages as described by Arksey and O’Malley [28] and extensions developed by Levac [29]. The five stages of the review process include stage 1: identifying the research question; stage 2: identifying relevant studies; stage 3: selecting studies; stage 4: charting the data; and stage 5: collating, summarizing, and reporting the results. Stage 6 involves a consultation exercise to inform and validate the review findings with critical stakeholders, which is optional. In this review, we conducted stages 1–5 of the given framework. For reporting the scoping review, the Preferred Reporting Items for Systematic Reviews and Meta-Analyses Extension for Scoping Reviews (PRISMA-ScR) checklist was followed [30].

### Search strategy

During August 2024, literature search for relevant research articles was performed via electronic search engines such as Scopus, PubMed, Embase, and the Cochrane while considering specific inclusion and exclusion criteria. The employed keyword combination and Boolean operators in each of the databases are mentioned in Table 2.

**Table 2:** Search terms used during the study

Pubmed	(Photobiomodulation OR low level laser therapy OR photomodulation OR red light OR phototherapy) AND (radiotherapy OR radiation therapy) AND radiodermatitis AND cancer;
Scopus	(TITLE-ABS-KEY (photobiomodulation) OR TITLE-ABS-KEY (low level AND laser AND therapy) OR TITLE-ABS-KEY (phototherapy) OR TITLE-ABS-KEY (red AND light) OR TITLE-ABS-KEY (photomodulation) AND TITLE-ABS-KEY (radiation AND therapy) OR TITLE-ABS-KEY (radiotherapy) AND TITLE-ABS-KEY (radiodermatitis) AND TITLE-ABS-KEY (cancer))
Embase	(photobiomodulation:ti,ab,kw OR 'low level laser therapy':ti,ab,kw OR photomodulation:ti,ab,kw OR phototherapy:ti,ab,kw OR 'red light':ti,ab,kw) AND (radiotherapy:ti,ab,kw OR 'radiation therapy':ti,ab,kw) AND radiodermatitis:ti,ab,kw AND cancer:ti,ab,kw
Cochrane	Photobiomodulation in Title Abstract Keyword AND "low level laser therapy" in Title Abstract Keyword AND radiotherapy in Title Abstract Keyword AND radiodermatitis in Title Abstract Keyword AND "Cancer" in Title Abstract Keyword

### Eligibility criteria

The inclusion and exclusion criteria used for the literature search are listed in Table 3.

**Table 3:** The inclusion and exclusion criteria considered for the selection of articles

Inclusion criteria	Exclusion criteria
• Study involves PBMT for the management of radiodermatitis • Cancer studies involving human subjects • Dermatitis caused only by radiation therapy • Original research articles including clinical trials, case reports, and pilot studies available in English language or English translation are available	• Studies used other than PBMT for the treatment of radiodermatitis • Review articles, book chapter, comments/commentary, letter to editor, conference proceeding, editorial, or unpublished data

### Data extraction and analysis

Primary screening was performed on the basis of the title and abstract of the articles by two independent authors (DR and CND) following the full-text screening of the articles to ensure their fulfillment of the inclusion criteria. A third evaluator (VP) was consulted to finalize the study selection. The items included in the data extraction sheet were (a) authors detail, (b) year of publication, (c) type of study, (d) radiation dose, (e) PBMT treatment plan, (f) RD grading scale, and (g) outcomes. The data extraction form was piloted by two reviewers before the study, to ensure the inclusion of all necessary details. Furthermore, the extracted data are summarized in tables which are helpful for comparing different laser parameters and corresponding outcomes, along with a narrative summary.

## Results

**Fig. 2:**
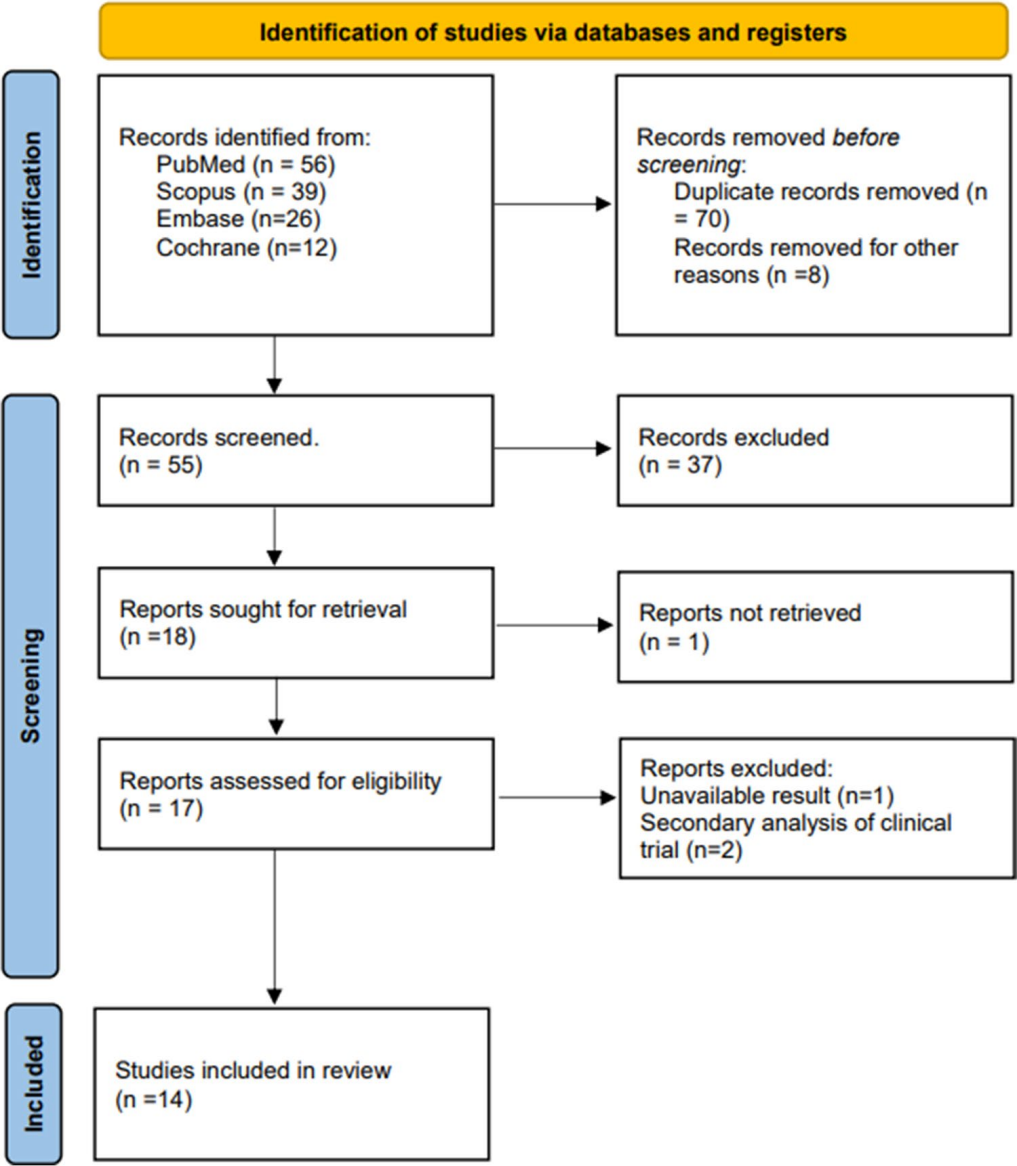
Search strategy employed according to the PRISMA flowchart

Using the previously reported keywords in Scopus, PubMed, Embase, and Cochrane Library, a total of 133 articles were obtained. Among these articles, 70 were excluded entirely because of duplication and eight were excluded for other reasons (e.g., comments, response letters to the editor). Furthermore, 37 records were excluded because the title and abstract did not match the inclusion criteria, and the full texts of the remaining 18 studies were assessed; one study was not accessible. Among the 17 papers, two were secondary analysis of clinical trials and one study was incomplete, resulting in the unavailability of complete results. Thus, 14 papers were ultimately selected for scoping review because they fulfilled the set inclusion criteria. Figure 2 depicts the search strategy utilized for the review report according to the PRISMA guidelines.

*NCI* National Cancer Institute, *RTOG* Radiation Therapy Oncology Group, *CTCAE* Common Terminology Criteria for Adverse Events, *RISRAS* Radiotherapy-Induced Skin Reaction Assessment Scale.

The pathobiology of RD includes direct tissue damage and the recruitment of inflammatory cells in response to skin irradiation which in turn damages epidermal and endothelial cells. Furthermore, free radicals released due to radiation induced DNA damage, along with inflammatory cytokines, can cause ulceration and other clinical abnormalities such as erythema. The intensity of skin reactions is influenced by a number of radiation parameters, including the overall treatment period, dose per fraction, type and energy of the beam, and total amount of radiation exposed to the skin [46].

Table 4 summarizes the outcomes of the studies related to the applicability of PBMT in treating RD. The 14 studies considered were of different study types, analyzing the effect of PBMT mainly on breast cancer (BC), head and neck cancer (HNC), and other cancer types, such as cervical and anal cancer. The study types included randomized controlled trials (35.71%), case reports (21.42%), prospective studies (14.28%), single institution analysis (14.28%), pilot studies (7.14%), and case series (7.14%). The influence of PBMT has been extensively studied in BC followed by HNC. RTOG and CTCAE are the commonly used scale to assess treatment efficacy of PBMT.

**Table 4:** Photobiomodulation therapy for treating radiation dermatitis in cancer patients: Summary of the outcomes

Study type	Sample size and geographic location of the study	Radiation dose	PBMT treatment	Additional skincare regime	Grading scale	Outcome	Comment	References
Breast cancer								
Prospective study	Treatment, *n* = 19 Control, *n* = 28 USA	5040 cGy	Total = 33–38 session; administered within 1 h of RT	Aquaphor was applied 3–4 times a day	NCI criteria	Positive; Treatment group 7 (36.8%)—Grade 0 reaction; 11 (57.9%)—Grade 1 reaction; 1 (5.3%)—Grade 2 reaction; None—Grade 3 reaction Control group None—Grade 0 reaction; 4 (14.3%)—Grade 1 reaction; 18 (64.3%)—Grade 2 reaction; 6 (21.4%)—Grade 3 reaction	Small sample size; included only post lumpectomy patients	DeLand et al. [31]
Randomized controlled, double-blind study	Treatment, *n* = 18 Control, *n* = 15 USA	45 to 50.4 Gy on chest wall 60.4 to 61.2 Gy on lumpectomy cavity	Total = 25/28 session; before and after RT	Aquaphor was applied 3–4 times a day	NCI criteria	No significant effect; Treatment group none—Grade 0 reaction; 6 (33.3%)—Grade 1 reaction; 12 (66.6%)—Grade 2 reaction; none—Grade 3 or higher reaction; Control group 1 (6.6%)—Grade 0 reaction; 4 (26.7%) Grade 1 reaction; 9 (60.0%)—Grade 2 reaction; 1 (6.6%)—Grade 3 reaction		Fife et al. [32]
Pilot study (DERMIS trial)	Treatment, *n* = 38 Control, *n* = 41 Belgium	66 Gy	Total = 6 sessions; along with RT (2 times/week)	Application of topical, hydroactive colloid gel 3 times a day (Flamigel®) In case of pain, silicone dressing was used	RTOG RISRAS	Positive; Treatment group 37 (97.4%) Grade 1 reaction; 1 (2.6%) Grade 2 reaction; None—Grade 3 reaction; Control group 29 (70.7%) Grade 1 reaction; 12 (29.3%) Grade 2 reaction; None—Grade 3 reaction		Censabella et al. [33]
Single-institution analysis	Treatment, *n* = 25 Control, *n* = 45 Germany	50.4 Gy	20–30 min prior to RT (2 times/week)	Palmitoylethanolamide cream for RD grade 1 and a phenol-methanal-urea-polycondensate cream for grade 2 RD	CTCAE 4.0	Positive; Treatment group 22 (88%)—Grade1 reaction; 3 (12%)—Grade2 reaction; None—Grade 3 reaction; Control group 25 (55.6%)—Grade 1 reaction; 18 (40%)—Grade 2 reaction; 2 (4.4%)—Grade 3 reaction		Strouthos et al. [34]
Randomized, placebo-controlled trial (TRANSDERMIS Trial)	Treatment, *n* = 60 Control, *n* = 60 Belgium	66 Gy	Total = 14 sessions; along with RT for 7 weeks (2 times/week)	Application topical, hydroactive colloid gel 3 times a day (Flamigel®) In case of pain silicone dressing was used	RTOG RISRAS	Positive; Treatment group 56 (93.3%)—Grade 1 reaction; 4 (6.7)—Grade 2 reaction; None—Grade 3 reaction; Control group 42 (70%)—Grade 1 reaction 16 (26.7%)—Grade 2 reaction; 2 (3.3%)—Grade 3 reaction		Robijns et al. [35]
Randomized multicentric clinical trial (LABRA trial)	Treatment, *n* = 39 Control, *n* = 32 Belgium	42.56 Gy	Total = 10 sessions; After RT for 5 weeks (2 times/week)	Application of topical, hydroactive colloid gel 3 times a day (Flamigel®) In case of pain, silicone dressing was used	RTOG	No significant effect; Treatment group 35 (90%)—Grade 1 reaction; 4 (10%)—Grade 2 reaction; None—Grade 3 reaction; Control group 23 (72%)—Grade 1 reaction; 9 (28%)—Grade 2 reaction; None—Grade 3 reaction	Small sample size	Robijns et al. [37]
Head and neck cancer								
Randomized controlled trial	Treatment, *n* = 30 Control, *n* = 30 China	Not reported	10 min, 2 times/day after RT for 6 weeks	None	RTOG	Positive; Treatment group 18 (60%)—Grade 0–1 reaction; 12 (40%)—Grade 2 reaction; None—Grade 3 reaction; Control group 2 (6.67%)—Grade 0–1 reaction; 19 (63.33%)—Grade 2 reaction; 9 (30%)—Grade 3 reaction	Lack of detail on the RT and PBMT parameters analyzed in the study	Zhang et al. [27]
Single-institution pilot study	Treatment, *n* = 33 Republic of Korea	60.39 Gy	Average = 14.97 sessions (3 times/week)	The exposed area received more than twice daily applications of topical moisturizer	CTCAE	Positive; 19 (57.6%)—Grade 1 reaction; 3 (9.1%)—Grade 2a reaction; 8 (24.2%)—Grade 2b reaction; 3 (9.1%)—Grade 3 reaction	Smaller sample size No control group considered	Park et al. [39]
Randomized, placebo-controlled trial (DERMISHEAD trial)	Treatment, *n* = 28 Control, *n* = 18 Belgium	30 × 2 Gy was delivered to the boost region and 30 × 1.8 Gy to the bilateral elective nodes	Total = 14 sessions; Following RT for a duration of 7 weeks (two times/week)	Application of topical, hydroactive colloid gel 3 times a day (Flamigel®^)^ In case of pain, silicone dressing was used	NCI-CTCAE v4.03 RISRAS	Positive; Treatment group 20 (71%)—Grade 1 reaction; 8 (29%)—Grade 2 reaction; None—Grade 3 reaction; Control group 4 (22%)—Grade 1 reaction; 11 (61%)—Grade 2 reaction; 3 (17%)—Grade 3 reaction	Low adherence rate of the participants in study	Robijns et al. [40]
Case series	Treatment, n = 15 Brazil	Total = 33 sessions	Group1: Before RT treatment Group 2: After RT treatment (5 times/week)	None	General grading ARD (Grading scale not specified)	Positive; PBMT for prevention of RD Group 1 6 (40%)- No RD 3 (20%)-Grade 1 3 (20%)- Grade 3 PBMT for healing RD Group 2 3 (10%)- RT interruption	As it is case series, minimization of margin of error and methods to increase precision has not been incorporated	Aires et al. [41]
Breast and head and neck cancer								
Prospective study	Treatment, *n* = 72 France	40 Gy	5 sessions/week carried out right before or after RT	If the patient had lesions, standard local treatment, analgesics, and corticosteroids were permitted, along with other therapy deemed essential for the patient’s well-being	NCI CTCAE v4	Positive; No adverse effect reported on the device CareMin650 Reduction in the severity of RD	No control group included	Bensadoun et al. [42]
Anal and cervical cancer								
Case report	Treatment, *n* = 2 Italy	Case 1 36 Gy (20 fractions) in pelvis, and additional 9 Gy on the anal canal Case 2 45 Gy	Case 1 Once every 2 days Case 2 Every other day for 2 weeks	None		Positive; Reduction in pain, bleeding, and itching, with no reported relapse of RD	In addition to the RT, chemotherapy was also administered as a combined regime	Gobbo et al. [43]
Case report	Treatment, *n* = 2 Brazil	Case 1 56 Gy Case 2 66 Gy	Case 1 6 sessions, Case 2 9 sessions	1% silver sulfadiazine and betamethasone valerate	RTOG	Positive; Reduction of RD from grade 3 to grade 1, with decrease in discomfort and pain	In addition to the RT, chemotherapy was also administered as a combined regime	Rocha et al. [44]
Case report	Treatment, *n* = 1 Brazil	5400 cGy (30 fractions) 5-week treatment	2 times a week with an interval of 48 h between sessions for 4 weeks	None	RTOG	Positive; Reduction of RD from grade 3 to grade 2, along with decrease in discomfort and pain		Hottz et al. [45]

Among the 14 research investigations analyzed, with the exception of two studies, [32, 37] the remaining studies demonstrated favorable results regarding the efficacy of PBMT in the treatment of radiation-induced dermatitis in individuals diagnosed with cancer. In studies with positive outcomes, the PBMT has been observed to be beneficial in reducing the severity of the RD. PBMT application has been studied as a preventive measure for the development of RD (35.71%), for the treatment and management of RD severity (50%), and for both the prevention and cure of RD (14.29%).

The Censabella and Robijns groups have extensively studied PBMT (808 and 905 nm) based management of RD in breast cancer. They began their research with pilot study (DERMIS trial) [33], whose results provided sufficient positive outcomes to conduct the TRANSDERMIS trial, which had a larger sample size and provided a definitive beneficial effect of PBMT in treating RD [35]. A retrospective study of the TRANSDERMIS trial patient population was conducted which ensured the long-term safety of the technique with no locoregional recurrence or new tumor formation [38]. Furthermore, the applicability of PBMT on patients undergoing hypofractioned whole-breast irradiation was not significant based on the basis of the LABRA trial results [37]. The DERMISHEAD trial focused on managing the RD developed in head and neck patients, and the results supported the implementation of PBMT among cancer patients [40].

### PBMT parameters

Table 5 summarizes the PBMT parameters used in the studies. The light sources used in the reported studies included LEDs (35.7%) and diode lasers (57.14%), with wavelengths ranging from red to near infrared light, i.e., wavelengths from 590 to 905 nm. The PBMT treatment included either a single laser wavelength (35.71%) or a combination of wavelengths (64.29%). The reported laser mode ranges from pulsed mode (21.42%), continuous mode (42.85%), and continuous pulsed wave mode (28.57%). Compared with classical laser therapy, multi-wave locked system (MLS) laser therapy emits lasers in continuous pulsed wave mode, which is considered to be more beneficial, as continuous lasers used to reduce inflammation whereas pulsed lasers used to induce analgesic effects [36]. Furthermore, irradiances range from 44.6 to 168 mW/cm^2^ and fluences ranging from 3 to 67 J/cm^2^ have been reported. Depending on the scanner, a contact or noncontact application method was used. Notably, the study by Zhang et al. fails to mention the complete details of the PBMT parameters [27], whereas other studies lacked specific details of power, fluence, and beam area. The power mentioned in the studies are variable as some have given the peak power alone, while others have average power. Thus, it is necessary to provide complete details of the PBMT parameters for comparison of the studies and to understand the efficiency of treatment.

**Table 5:** Details of the photobiomodulation parameters used in the management of RD

PBMT type	Wavelength (nm)	Operating mode	Irradiance (W/cm^2^)	Fluence (J/cm^2^)	Power (W)	Beam area (cm^2^)	Timing and anatomical location	Application technique	Reference
LED	590 nm	Pulsed mode	-	0.15 J/cm^2^	-	-	Entire breast region	In contact with the skin	DeLand et al. [31]
	590 nm	Pulsed mode					35 s	2 cm from the patient’s skin	Fife et al. [32]
	660 nm 850 nm (combination)	Pulsed mode	44.6 mW/cm^2^	0.15 J/cm^2^	1390 mW	-	Breast fold and axilla: 60 s		Strouthos et al. [34]
	590 nm and 830 nm ± 7 nm (combination)	Continuous mode	100 mW/cm^2^	60 J/cm^2^	-	-	11 min, neck region	Approximately 20 cm from the neck	Park et al. [39]
	650 nm	Continuous mode	Dermal pads-21 mW/cm^2^	3 J/cm^2^ 6 J/cm^2^	-	-	Dermal pads—2 min 23 s-3 J/cm^2^, 4 min 46 s-6 J/cm^2^	In contact with the skin	Bensadoun et al. [42]
Diode laser	808 nm 905 nm (combination)	Continuous pulsed wave mode	0.168 W/cm^2^	4 J/cm^2^	1.1 W 25 W (peak power)	19.635 cm^2^	Whole breast: 384 ± 93 s, inflammatory fold:120 ± 39 s axilla: 153 ± 41 s	5 cm above the skin	Censabella et al. [33]
	808 nm 905 nm (combination)	Continuous pulsed wave mode	0.168 W/cm^2^	4 J/cm^2^	3.3 W (average power)	19.625cm^2^	Whole breast: 420–720 s inframammary fold: 103 s axilla: 68 s	5 cm above the skin	Robijns et al. [35]
	808 nm 905 nm (combination)	Continuous pulsed wave mode	0.168 W/cm^2^	4 J/cm^2^	3.3 W (average power)	3.14 cm^2^	Whole breast: ± 420–720 s, inframammary fold: ± 103 s, axilla: ± 68 s	5 cm above skin	Robijns et al. [37]
	808 nm 905 nm (combination)	Continuous pulsed wave mode	0.168 W/cm^2^	4 J/cm^2^	3.3 W (average power)	3.14 cm^2^	300–600 s, head and bilateral neck region	5 cm above skin	Robijns et al. [40]
	970 ± 15 nm 660 ± 15 nm	Continuous mode	-	67.5 J/cm^2^ 45 J/cm^2^			Genital and anal area	Noncontact mode	Gobbo et al. [43]
	660 nm	Continuous mode	3.57 mW/cm^2^ 1.11 mW/cm^2^	35.71 J/cm^2^ 27.77 J/cm^2^	100 mW, 40 mW	0.028 cm^2^ 0.036 cm^2^	10 s/point and 25 s/point cervical region except the thyroid region	In contact and perpendicular with skin	Rocha et al. [44]
	660 nm ± 10 nm	Continuous mode	187.5 mW/cm^2^ for the first 360 s, and 375 mW/cm^2^ for the last 120 s		100 mW		Perianal area and anal region	Laser attached to spacer which is in contact with skin	Hottz et al. [45]
	660 nm ± 10 nm (individual and combination) 808 nm ± 10 nm (combination)	Continuous mode	3.57 W/cm^2^	70 J/cm^2^, 140 J/cm^2^, and 210 J/cm^2^	100 mW	0.028 cm^2^	Neck region	In contact with the skin	Aires et al. [41]
Red light phototherapy	-	-	-	-	-	-	10 min	15–20 cm from the wound surface	Zhang et al. [27]

### Effect of PBMT on pain, RT interruption, and quality of life (QoL)

In addition to the effects of PBMT on the severity of RD, other important parameters, such as pain level, RT interruption, and QoL have also been reported. Approximately six studies have evaluated pain levels via the visual analog scale (VAS), or the NCI-5 point scale for grading skin reaction questionnaire and the numerical rating scale. Among them, two studies were performed on breast cancer patients; study by Strouthos et al. reported that 60% of the treatment group reported no pain, whereas in the control group only 28.9% reported no pain with the rest reporting pain intensity up to VAS-5 scale [34]. In contrast, the study by Fife et al. revealed no difference in pain level after PBMT treatment [32]. Zhang et al. reported pain reduction in the HNC patients’ treatment group, whereas increased pain was observed in the control group after each RT [27]. A study by Bensadoun et al. investigated pain intensity in both BC and HNC patients and observed significant pain reduction as 87.5% reported no pain [47]. Two case studies on anal cancer patients also reported reduction in pain intensity over the course of treatment [43, 45]. On the basis of these studies, we have greater confidence in the application of PBMT as an analgesic in the treatment and management of RD.

Interruption of the RT treatment plan due to severe skin reactions is considered another major setback. Among the reported studies, four reported RT interruption. Deland et al. reported that 5.3% of patients in the treatment group and 67.9% of those in the control group experienced RT interruption [31]. Fife et al. reported that 11.1% and 6.7% of sample population discontinued RT from the treatment and control groups respectively [32]. Strouthos et al. reported no RT interruption in the treatment group, but 4.4% of the control participants discontinued the treatment [34]. Aires et al. reported that 1% of samples discontinued RT due to RD [41]. Furthermore, a patient’s quality of life (QoL) during treatment plays a very important role in patient satisfaction and continuation of treatment. Three studies evaluated the QoL of patients on the basis of the skindex-16. Based on these findings, only one study reported improvement in QoL [35], whereas the other two studies reported no significant improvement in the PBMT group [33, 40].

### Safety of PBMT

There has been a debate about the possible tumor promoting effect of PBMT due to residual cancerous cells. An in vivo study investigating the PBMT effect on melanoma reported induction of tumor growth leading to angiogenesis [48]. However, another study reported PBMT to be safe for amelanotic non-pigmented melanoma, whereas for melanotic pigmented cells the PBMT triggers different responses depending on the light parameters, i.e., NIR laser at lower dose was observed to be promoting the cell invasiveness, whereas red light reduced the cell invasiveness [49]. Further, potential interference of PBMT in the anti-cancer treatment plan has also been one of the concerns. Recent study involving orthotopic animal model bearing tumor has further investigated these claims, wherein PBMT followed by radiation therapy did not reduce the efficiency of the RT in killing tumor cells [50]. The PBMT-related risk assessed in the studies reported no adverse effects either on RD severity or the cancer reoccurrence during the course of the study. [32, 37] Even among the two studies with no significant outcome, the one that presented no adverse effects rather failed to present meaningful outcomes compared to the control group [32, 37]. Long-term follow-up (5 years after end of RT) was conducted for the TRANSDERMIS study population. The study reported no significant variation in disease free survival (73.7% vs. 98.3%), cancer free survival (68.4% vs. 77.8%), and overall survival (87.9% vs. 98.3%) between control and treatment group, thus, suggesting that the PBMT treatment did not elicit tumor recurrence over an extended period of time [38]. However, this is the only study that has conducted long-term follow-up, so more studies on long-term effects on cancer reoccurrence and tumor development are needed to confirm the safety of PBMT.

### Skincare regime and PBMT

The skincare regime plays a major role in managing skin reactions and maintaining the skin barrier after undergoing RT, thereby reducing the severity of RD. During RT, the common skin issues include erythema, dryness, and hyperpigmentation. A secondary analysis of the TRANSDERMIS study population has revealed that the biophysical skin measurements were mainly moist desquamation which was significantly reduced in the treatment group that underwent PBMT. In addition, large breast volume has been reported to be a risk factor for the development of moist desquamation [34]. In addition to the benefits from PBMT, most studies have suggested the institutional skincare protocols for patients, with the focus on maintaining the cleanliness of the area along with the regular application of topical agents to counter irritation. The commonly recommended topical agents in this area include Aquaphor, a petroleum-based emollient [31, 32], Flamigel®, a hydroactive colloid, Mepilex®, a silicone dressing [33, 35–37, 40], Palmitoylethanolamide cream, phenol-methanal-urea-polycondensate cream [34], and 1% silver sulfadiazine, an antibiotic and betamethasone valerate, a steroid agent [44]. Among these agents, few have been proven to be effective individually in the management of RD. Flamigel® has been shown to be beneficial in reducing pain and soothing effects; however, no effect on erythema has been reported [51]. Similarly, silver sulfadiazine and betamethasone valerate have also been shown to reduce the severity of RD [52, 53], and Mepilex Lite dressings are known to accelerate wound healing in RD patients [54]. Thus, the PBMT results of the studies that have included these agents in the skincare protocol could be the cumulative effect of PBMT and the topical agent rather than PBMT alone. These findings provide us with the possibility of enhancing the PBMT effect with topical agents that have been proven to be effective in treating RD such as timolol and Biafne® [55, 56].

### PBMT and RD: advancements

The varied results obtained from these clinical studies can be explained only by a better understanding of the mechanism of action of PBMT on RD. Park et al. used a mouse model to evaluate the effect of PBMT (633 nm, 830 nm) on RD and reported that PBMT was able to reduce the severity of RD by reducing inflammation and dermal damage, but no significant difference was observed between the beneficial effects of 633 nm and 830 nm [57]. Another study on a rat model using an LED-based PBM apparatus for RD observed the best results in the treatment group with a combination of 630 nm + 850 nm wavelengths, as well as in the group receiving 630 nm alone, based on macroscopic evaluation, i.e., RTOG grading of the wound site by investigators. Further confirmation done via gene expression analysis revealed that both tumor necrosis factor (TNF-α) and interleukin-10 levels were lower than those in the control group [58]. In radiation induced skin reaction, the NF-Kb pathway is activated along with the production of many pro-inflammatory cytokines such as cyclooxygenase (COX), TNF-α, and chemokines; thus, inhibition of these can improve skin tolerance to RT [59]. It is possible that the impact of PBMT on RD follows a similar mechanism as previous studies have reported that PBMT has an anti-inflammatory effect through the inhibition of prostaglandin E2 (PGE2), COX-2, and TNF- α [58, 60].

There has also been the development of efficient PBMT devices that could make this treatment option more reliable and effective for clinical use. CareMin650 is one such device that uses LED emission (650 nm) on the surface in contact with either a derma pad or an oral pad on the basis of the site of adverse reaction mode and has reported promising results in treating RD in both HNC and BC patients. Thus, further standardization of PBMT parameters could be easily implemented in the treatment population [47]. Considering the success of these PBMT treatments in HNC and BC, this approach has also been applied in treating RD in other cancers. A case report on PBMT for lesions due to radiation therapy targeting anal canal treatment reported a reduction in the RD grade along with associated symptoms such as pain and burning sensations [45]. In another study that focused on two patients with acute cervical RD, a reduction in RD grade after RT with enhanced healing was reported [44].

## Conclusion and future prospects

PBMT can be considered a reliable and effective treatment modality for reducing the severity of RD. However, detailed studies related to the long-term effects of PBMT and its effect on pain intensity and quality of life (QoL) will aid in better assessment of the technique. Few studies conducted lack details on PBMT parameters and the outcomes, which needs to be rectified in future studies. The heterogeneity in the reported laser parameters, the use of varying grading scales for RD assessment, and incomplete reporting of laser parameters are among the limitations of this review. Significant obstacles to the execution of robust statistical analyses are posed by these factors, which limit direct comparison of outcomes. For better applicability and benefits for the patient population, a standardized protocol specifying the ideal frequency and timing of PBMT on the basis of the severity of RD is essential. Recent studies have suggested towards the possible combination treatment options such as topical agents applied alongside PBMT to enhance the effectiveness of the treatment. Further, a better understanding of the mechanism of action of PBMT on RD needs to be investigated on animal models with combination of wavelengths. In addition, patient reported outcomes and the cost effectiveness of the treatment need to be considered in future studies. Addressing these research gaps could contribute to a more robust and evidence-based approach to the application of PBMT in the treatment of RD, ultimately improving patient outcomes and the quality of care.

## Data Availability

No datasets were generated or analysed during the current study.
